# Conservative Treatment of Spontaneous Rectus Sheath Hematomas: Single Center Experience and Literature Review

**DOI:** 10.1155/2019/2406873

**Published:** 2019-02-21

**Authors:** Banu Karapolat, Halil Afsin Tasdelen, Hatice Ayca Ata Korkmaz

**Affiliations:** ^1^Department of General Surgery, Kanuni Training and Research Hospital, Trabzon, Turkey; ^2^Department of Radiology, Kanuni Training and Research Hospital, Trabzon, Turkey

## Abstract

**Introduction:**

Spontaneous rectus sheath hematoma (SRSH) is characterized by bleeding within the rectus abdominis muscle sheath, one of the rare causes of acute abdominal pain. Early diagnosis is imperative in SRSH to prevent complications and the treatment is usually conservative. We intended to present in this study our experience with SRSH patients with respect to diagnostic evaluation and management of their disease.

**Materials and Methods:**

In this retrospective study, 14 patients who had received treatment for SRSH in our clinic between January 2012 and December 2017 were assessed in terms of demographic and clinical characteristics, comorbidities, laboratory parameters, diagnostic approach methods, treatment practices, length of hospital stay, and patient outcomes.

**Results:**

The patients consisted of 10 (71.4%) females and 4 males (28.6%). The age of the patients ranged between 47 and 93 with a mean age of 66.5 ± 12.1. Anticoagulant treatments were being administered to 5 (35.7%) patients, antiplatelet treatments to 4 (28.5%) patients, and both anticoagulant and antiplatelet treatments to 4 (28.5%) patients. The most common triggering factor was severe cough and the most common initial symptom acute abdominal pain (71.4%). In physical examinations, the entire patients had generalized abdominal tenderness, 10 (71.4%) voluntary guarding and 7 (50%) a right lower quadrant mass. The diagnosis was confirmed by abdominal ultrasonography and computed tomography. Based on the computed tomography findings, the disease was classified as Type 2 found in 9 (64.3%) patients, Type 1 in 3 (21.4%) patients, and Type 3 in 2 (14.2%) patients. All the patients were treated conservatively. They were hospitalized for 1 to 23 days. There was no mortality. All the patients were followed up between 3 months and 2 years and no recurrence was recorded.

**Conclusion:**

Considering the presence of SRSH particularly in older female patients who use anticoagulant drugs and have newly developed an abdominal pain and a palpable mass after coughing spells is the key to make an early and correct diagnosis and to prevent possible morbidity and mortality with an appropriate treatment method.

## 1. Introduction

Spontaneous rectus sheath hematoma (SRSH) is a benign pathology developing after accumulation of blood in the rectus sheath due to a rupture of epigastric vessels or tear of the rectus muscle [1]. Considered as a complication of patients who receive anticoagulants, unfractioned and low-molecular-weight heparins (LMWH) and/or antiplatelet therapies and as a relatively rare cause of acute abdominal pain, this clinical condition can usually be self-limiting when conservative treatment methods are used [2, 3]. However, SRSH can sometimes progress fast and reach life-threatening magnitudes and may even result in hemorrhagic shock and death due to massive bleeding [4, 5]. Its major risk factors include female gender, advanced age, hypertension, atherosclerosis, hematologic diseases, collagen vascular disorders, degenerative muscle diseases, intra-abdominal injections, paracentesis, peritoneal catheter insertion, pregnancy, obesity, blunt trauma, abdominal surgery, excess-uncontrolled exercising, and increased abdominal pressure from cough or sneeze [6].

Clinical, laboratory findings and radiological examinations are helpful in diagnosing SRSH. As anticoagulant therapies are the top predisposing factor in its etiology, spontaneous hematoma has become increasingly common in recent years with increase in the number of patients receiving these treatments. Its symptoms are generally nonspecific, often involving abdominal swelling, tenderness and pain, fever, nausea, vomiting, guarding, rebound, tachycardia, fatigue, hypotension, and syncope. In physical examinations, mostly a painful, nonpulsatile mass (often localized in the right lower quadrant), tenderness, and abdominal guarding are detected through palpation [7]. Berna et al. proposed in their study conducted in 1996 a method for classification of rectus sheath hematoma based on computed tomography (CT) findings. This method divides hematomas into 3 types depending on presentation, severity, prognosis, and treatment. Type 1 is a mild, intramuscular, and unilateral condition without any hemodynamic compromise and does not require hospitalization. Type 2 is a moderate, unilateral or bilateral, and intramuscular condition with blood between rectus abdominis and transversalis fascia with a possible decrease in hematocrit and requires hospitalization. Type 3 is a more severe, unilateral or bilateral condition usually affecting patients taking anticoagulants where blood extends up to peritoneum and prevesical space with a possible decrease in hematocrit and a hemodynamic impairment and requires hospitalization and blood transfusion [8]. The treatment varies depending on the severity, location, and size of the hematoma, the patient's condition, the degree of hemodynamic impairment, the status of coagulation, and the type and extent of SRSH as found with tomography.

In this study, we tried to present the demographic and clinical characteristics, comorbidities, laboratory parameters, hospitalization times, and outcomes of the patients which we monitored and treated due to SRSH diagnosis as well as our diagnostic approaches and treatment practices, also referring to the information in the literature.

## 2. Materials and Methods

### 2.1. Subjects and Study Protocol

We reviewed retrospectively 14 patients who were diagnosed and treated for SRSH at the General Surgery Clinic in Trabzon Kanuni Training and Research Hospital between January 2012 and December 2017. The recorded data included demographic characteristics, anamneses, comorbid diseases, symptoms, physical examination findings, laboratory test results such as hemoglobin, international normalized ratio (INR), prothrombin time (PT), activated partial thromboplastin time (aPTT), hematoma sizes, SRSH types per tomography findings as well as the treatment methods used, previous anticoagulant and/or antiaggregant therapies patients used and the reasons for starting such therapies, the time to restarting these therapies after recovery, the amount of blood and blood products given, lengths of hospital stay and long-term follow-up results.

The protocol of this study was approved by the local ethics committee and it was implemented in accordance with the principles of the Helsinki Declaration revised in 2000.

### 2.2. Statistical Analysis

All statistical data analyses were carried out using descriptive statistics on the SPSS statistics software (SPSS Inc., Chicago, IL, USA), version 15.0. Kruskal Wallis; Mann–Whitney U and Chi Square tests were used for comparisons. A Spearman correlation analysis was performed to evaluate the relationship between hematoma size and INR. The statistical significance level was accepted as p<0.05.

## 3. Results

From the 14 patients diagnosed with SRSH, 10 (71.4%) were female and 4 (28.6%) male. The ages of the patients ranged between 47 and 93 with a mean age of 66.5 ± 12.1. The anamneses of the patients revealed that 5 (35.7%) patients had excessive cough due to chronic obstructive pulmonary disease (COPD) attacks, 3 (21.4%) patients excessive cough due to flu, 2 (14.2%) patients excessive exercising, and 2 (14.2%) patients strain due to constipation. The mainly comorbid diseases included hypertension (HT) in 9 (64.3%) patients, obesity in 8 (57.1%), diabetes mellitus (DM) in 6 (42.3%), COPD in 5 (35.7%), coronary artery disease (CAD) in 5 (35.7%), chronic constipation and dyspepsia in 4 (28.5%), and atrial fibrillation (AF) in 3 (21.4%). The mainly initial symptoms included sudden abdominal pain in 10 (71.4%) patients, hypotension in 3 (21.4%), vomiting in 2 (14.2%), and ecchymosis in abdominal wall in 2 (14.2%).

Six (57.1%) patients were using insulin due to DM and 3 (21.4%) patients subcutaneous LMWH. All of these patients were using their upper arm regions for injections.

In their physical examinations, the entire patients had generalized abdominal tenderness, 10 (71.4%) of them had voluntary guarding, 7 (50%) had a right lower quadrant mass, and 3 (21.4%) had bilateral lower quadrant masses.

The hemoglobin values of the patients ranged between 8.1 and 15.7 g/dL (mean 12.9) and their INR values between 0.8 and 5.5 (mean 2.9). There was a strong positive correlation between INR and hematoma size (r = 0.714, p = 0.004). PT ranged between 9.8 and 88.9 sec. with a mean time of 36.1 sec. aPTT ranged between 26.4 and 192 sec. averaging 87 sec.

**Figure 1:**
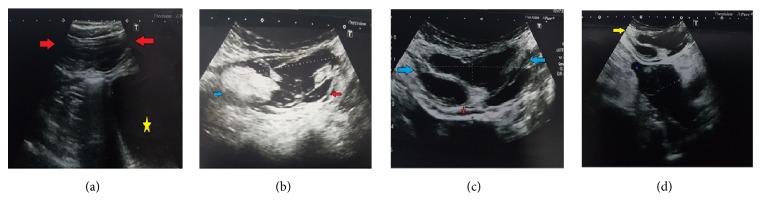
(a) B mode ultrasonographic image shows the SRSH. Increased muscle size with an ovoid fusiform border (red arrows) is seen. The SRSH is unilateral and does not dissect the fascial planes. Normal bladder is shown with yellow star (Type 1). (b) B mode ultrasonographic image shows that the SRSH is intramuscular but with blood between the muscle and the transversalis fascia, bilaterally. No blood was observed in the prevesical space. Complicated part of the SRSH is shown with blue arrow. Blood between the transversalis fascia and the muscle is shown with a red arrow (Type 2). (c) In the ultrasonographic image, the SRSH affects the muscle (blue arrows), and the blood is seen between the transversalis fascia and the muscle (red star) (Type 3). (d) In the ultrasonographic image, the SRSH affects the muscle (yellow arrow), and the blood is seen in the peritoneum and perivascular space (purple star) (Type 3).

All patients underwent an abdominal ultrasound (USG) scan and hypoechoic masses compatible with hematoma were detected in the right or left rectus sheaths. The sizes of hematomas ranged between 12 mm and 97 mm. The mean hematoma size was 22.3 ± 10 mm in Type 1, 40.6 ± 9.6 mm in Type 2, and 93.5 ± 4.9 mm in Type 3. There was a statistically significant difference between hematoma sizes with respect to their types (p = 0.014) (Figures 1(a), 1(b), 1(c), and 1(d)).

**Figure 2:**
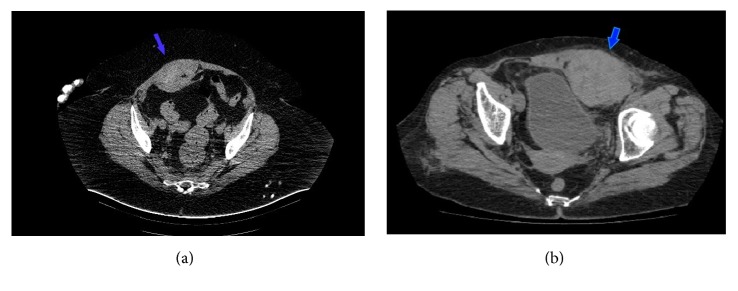
(a) Axial computerized tomography image shows Type 2 right SRSH that mimics an extra-abdominal mass image (blue arrow). (b) Axial computerized tomography image shows Type 3 left SRSH with prevesical fossa involvement (blue arrow).

An abdominal CT scan was administered to the entire patients and the tomographic data showed that 9 (64.3%) patients had Type 2 SRSH, 3 (21.4%) patients Type 1 SRSH, and 2 (14.2%) patients Type 3 SRSH (Figures 2(a) and 2(b)).

While 5 (35.7%) patients were receiving anticoagulant therapy, 4 (28.5%) antiplatelet therapy, and 4 (28.5%) both anticoagulant and antiplatelet therapies, 1 (7.1%) patient was not using any drug of this group. Of these patients, 9 (64.3%) were using these drugs due to cardiac reasons, 2 (14.2%) due to deep vein thrombosis (DVT), 1 (7.1%) due to cerebrovascular disease (CVD), and 1 (7.1%) due to pulmonary embolism (PE). After the patients had recovered clinically, they were reintroduced the abovementioned drugs 2-23 days (mean 7.6 days) later.

All the patients received medical treatment and 8 (57.1%) patients in total were given blood or blood products. Fresh frozen plasma (FFP) was given to 8 (57.1%) of these and red blood cells (RBC) were given to 3 (21.4%). Additionally, 5 (35.7%) patients were given vitamin K. There was no statistically significant correlation between SRSH type and need for blood transfusion (p=0.586). No interventional procedure or surgical treatment was administered to any of the patients.

The patients were bedded in the hospital for 1-23 days (mean 7.9 ± 6.4 days). The mean length of stay was 3.0 ± 2.6 days in Type 1, 6.8 ± 4.0 days in Type 2, and 20.0 ± 4.2 days in Type 3. There was a statistically significant difference between the lengths of stay with respect to SRSH types (p = 0.035). There were no thromboembolic complications or mortality. All the patients were followed up for 3 months to 2 years and their physical examinations were performed during their check-ups. No recurrences were found in any of the patients. All of these abovementioned demographic and clinical characteristics of the patients are summarized in Table 1.

## 4. Discussion

SRSH occurs mostly in patients who receive an anticoagulant or antiaggregant therapy for some reason. Excessive strain occurring during severe coughing and constipation, increased abdominal pressure particularly in pregnancy and underlying bleeding disorders lead to SRSH as a result of a rupture in the superior or inferior epigastric vessels. While the bleeding occurring in the superior epigastric vessels is unilateral, minor, and fusiform and can be self-limiting, the bleeding occurring in the inferior epigastric vessels may grow gradually due to the absence of the rectus sheath on the posterior, gains a bilober character, and can expand beyond the midline heading towards the posterior. Such patients lose more blood in this way. When the anatomy of rectus sheath is examined, it is seen that the region is supported by the rectus sheath on the arcuate line at the posterior, but the supporters of the rectus sheath at the posterior are only transversalis fascia and peritoneum below the arcuate line, that is, in the region where the inferior epigastric vessels are located. Since these do not have any strong buffering property, hematomas below the arcuate line have a tendency to expand [9, 10].

The inferior epigastric artery is relatively more important than the superior epigastric artery in terms of blood circulation in the abdominal wall because the outer diameter of the deep inferior epigastric artery (DIEA) is 3.4 mm on the average, whereas that of the superior epigastric artery is 1.6 mm. Also, the largest periumbilical perforators take origin from the terminal branches of the DIEA and are often located within the lowest tendinous intersection of the rectus abdominis [11]. According to the law of Laplace, the smaller the radius, the less the wall tension. This means that diameter and wall tension are directly proportional [12]. For this reason, we think that a reason for SRSH to occur more frequently under the umbilicus is that the diameters of superior and inferior arteries are different. In this study, SRSH was detected in 8 (57.1%) patients under the umbilicus and in 6 (42.9%) patients above the umbilicus. Especially in older women vessels and muscles become more susceptible to trauma due to decreased muscle mass, making SRSH more common in this group. In fact, 71.4% of the patients in this study were women at advanced age.

In the anamneses of the patients diagnosed with SRSH, use of anticoagulant drugs is one of the most common risk factors. In our study, 92% of the patients had been receiving anticoagulant and/or antiaggregant therapy for various reasons. Such therapies not only impair coagulation procedure with their anticoagulant effects but also lead to thrombocytopenia, which results in SRSH. In a study on 115 rectus sheath hematoma cases made by Sheth et al., use of anticoagulant and antiaggregant drugs in combination was found to increase the risk of major bleeding and rectus sheath hematoma even further and 1/4 of the subjects in their study were found to use both drugs at the same time [13]. Similarly, we also found in our study that 28% of the patients used these two groups of drugs in combination. Additionally, there was a strong positive correlation between INR and hematoma size. Hematomas reaching very large sizes were seen in patients having excessively high INR values due to anticoagulant overdoses. This situation can be explained as bleeding that can occur in any part of the body at high INRs happens to take place in the rectus sheath in these patients.

As rectus sheath hematoma is found in less than 2% of patients presenting with acute abdominal pain, it does not readily come to mind as one of the initial diagnoses of acute abdominal pains and making the diagnosis clinically may be difficult especially in emergency rooms. SRSHs may be confused mostly with appendicitis, renal colic and abdominal hernia that can cause pain in the right lower abdominal quadrant as well as with gynecological diseases and other intra-abdominal pathologies such as diverticulitis, biliary diseases, pancreatitis, intestinal obstruction, colorectal diseases, abdominal tumors, and ruptured aortic aneurysm [1]. Although USG is used for initial examination due to easy access, fast application, and absence of radiation, contrast-enhanced CT is preferred as a primary diagnostic modality with 100% sensitivity and specificity in establishing a diagnosis, and it can even show active bleeding [14, 15]. CT also prevents unnecessary surgical interventions by excluding other intra-abdominal pathologies, provides information on the origin, extension, and nature of the hematoma, and helps in identification of the treatment option by classifying rectus sheath hematomas by their anatomic structures, sizes, and localizations. While small hematomas are treated using conservative methods such as bed rest, analgesia, ice application, compression, and treatment of underlying predisposing conditions, larger hematomas should be treated with fluid resuscitation, blood transfusion, and reversal of anticoagulation if the patient is not stable hemodynamically and there is expanding hematoma or symptomatic anemia. In such cases, anticoagulant and antiaggregant therapies need to be discontinued until the bleeding is controlled. In patients who do not respond to conservative treatment, are unstable hemodynamically and have uncontrollable bleeding or a hematoma continually increasing in size or an infected complicated hematoma and patients who show severe peritoneal irritation or signs of abdominal compartment syndrome, an angiography should be performed to identify the bleeding artery radiologically, coil or gel embolization should be applied to the bleeding vessel, or the hematoma should be drained surgically and the bleeding artery ligatured [16]. In their retrospective study involving 29 patients, Gradauskas et al. observed that 5 patients (17.2%) had the signs and symptoms of hypovolemic shock and 4 of them had to undergo embolization, which resulted successful in all of them [17]. It should be kept in mind that surgical intervention has a mortality risk and should be considered a technique of last resort [18]. In general, not all SRSHs relapse or leave sequels in the long-run. All of the patients in this study were treated conservatively and their hematomas were resolved spontaneously. Effective analgesia was provided and local ice application was done in all patients. Additionally, blood or blood products replacements were performed in 8 patients and 5 of these were also given subcutaneous vitamin K. Since there was no SRSH expansion in their follow-up ultrasonographies while staying in the hospital, their conservative treatment was continued. Interestingly, no statistically significant correlation was found in our study between the types of SRSH and need for blood transfusion. Blood or blood product replacements were done more in Type 2 and Type 3 cases where the magnitude of bleeding and hematoma were larger. We think the statistical result obtained here may have been influenced by the small size of the sample.

This study showed that there was an increase in hematoma size as the SRSH type advanced from 1 to 3. This is consistent with the literature data [16].

A statistically significant difference was found in our study between lengths of hospitalization with respect to SRSH types. The patients with Type 3 SRSH were observed to stay longer in the hospital than those with other types. We think that this was associated with the fact that Type 3 involved more bilateral bleeding extending to the fascial plan, inner peritoneum, and prevesical region, which led to a drop in hemoglobin values and resulting prolongation of treatment time and hematoma resorption time.

Rarely, mortality can occur in SRSH cases (about 4% on the average) [5, 7]. In the literature, mortality seems to be associated more with serious comorbid diseases, anticoagulant therapies, broad SRSHs, increased number of blood transfusions, and upper gastrointestinal system bleedings [16, 19]. There was no mortality in the present study.

The limitations of this study include its retrospective nature and small sample size. The diagnosis, risk factors, and treatment methods for SRSH patients that have been described here cannot be generalized. Nevertheless, we think the data obtained here may become more valuable with further multicenter studies including a broader patient population.

In conclusion, SRSH is a cause of acute abdominal pain that has come to occur more frequently today due to an increase in the use of anticoagulant and/or antiaggregant drugs for various reasons. Any comorbid disease should immediately be questioned particularly in older female patients who use anticoagulant drugs, who have a newly developed abdominal pain and a palpable mass, who have low hemoglobin levels, and who have hypotension or tachycardia. Conditions that increase intra-abdominal pressure such as cough, sneeze, and straining should be investigated by way of a careful anamnesis. Suspecting of SRSH in such situations will enable early and correct diagnosis, and, with an appropriate treatment method, morbidity and mortality will be prevented in these patients before any hemodynamic instability, abdominal compartment syndrome, or multiple organ dysfunction syndrome occur.

## Figures and Tables

**Table 1:** Detailed information of patients with SRSH. F: female, M: male, HT: hypertension, DM: diabetes mellitus, MVR: mitral valve replacement, COPD: chronic obstructive pulmonary disease, CAD: coronary artery disease, CABG: coronary artery bypass grafting, AF: atrial fibrillation, AVR: aortic valve replacement, DVT: deep vein thrombosis, MI: myocardial infarction, PE: pulmonary embolism, CVD: cerebrovascular disease, LMWH: low-molecular-weight heparins, ASA: acetylsalicylic acid, RBC: red blood cells, FFP: fresh frozen plasma, INR: international normalized ratio, PT: prothrombin time, aPTT: activated partial thromboplastin time.

Patient No.	Gender	Age (year)	Comorbidity	Anticoagulant and/or Antiplatelet Treatment	Physical Examination	Symptoms	Hematoma Size (mm)	Types according to the Tomography Findings	Length of Hospitalization (day)	Begin Retreatment (day)	INR	PT (sn)	aPTT (sn)	Blood Transfusion	Hemoglobin (g/dL)
1	F	52	HT-DM- MVR-COPD	LMWH+ASA	Right lower quadrant mass	Abdominal pain	33	Type 2	5	5	1.1	13	28.4	-	13.6

2	M	47	CAD	-	Paraumbilical mass	Abdominal pain- Nausea	12	Type 1	1	-	1	12.1	26.4	-	15.7

3	F	81	AF-Constipation	Warfarin-ASA	Right lower quadrant mass	Abdominal pain	35	Type 2	4	4	5.5	66.9	171	1 U RBC, 1 U FFP and vitamin K	9.2

4	F	74	HT-AVR-Constipation- Obesity	Warfarin-ASA	Bilateral lower quadrant mass	Hypotension- Vomiting	97	Type 3	23	23	4.5	57.1	140	2 U RBC, 1 U FFP and vitamin K	8.1

5	F	63	DM-DVT story- Constipation- Obesity	LMWH	Left lower quadrant mass	Abdominal pain	52	Type 2	9	9	4.2	51.1	130	1 U FFP and vitamin K	12.7

6	M	67	HT-DM-CAD-Previous MI- Obesity	ASA	Right lower quadrant mass	Abdominal pain	32	Type 2	3	3	0.8	9.8	24.9	-	14.9

7	F	65	HT-CAD- Constipation	ASA	Right upper and lower quadrant mass	Abdominal pain-Fever	34	Type 2	3	3	0.9	10.9	28	-	15.0

8	F	78	MVR-HT-Obesity- CAD-Coronary stent	Warfarin-ASA	Right lower quadrant mass	Abdominal pain- Hypotension	47	Type 2	12	10	7	88.9	192	2 U FFP and vitamin K	11.4

9	F	61	PE-COPD- Obesity	Warfarin	Right lower quadrant mass	Abdominal pain	48	Type 2	11	11	3	36.5	93.3	1 U FFP	13.8

10	F	59	HT-AF- COPD-Obesity	Warfarin	Bilateral lower quadrant mass	Hypotension- Vomiting	90	Type 3	17	17	4.4	57.1	125	1 U RBC, 1 U FFP and vitamin K	8.8

11	F	56	HT-CAD- CABG	ASA	Right upper and lower quadrant mass	Ecchymosis	23	Type 1	2	2	1	12.1	31.1	-	14.3

12	M	71	HT-DM-CVD	Clopidogrel	Right lower quadrant mass	Ecchymosis	30	Type 2	3	3	0.9	10.3	26.4	-	15.5

13	M	93	HT-DM-AF- COPD-Obesity	Warfarin	Right lower quadrant mass	Abdominal pain	32	Type 1	6	4	2.5	30.4	77.7	1 U FFP	14.7

14	F	65	DM-Hospitalized with DVT- COPD-Obesity	LMWH	Bilateral lower quadrant mass	Abdominal pain-Sencop	55	Type 2	12	12	4	50	124	1 U FFP	13.9

## Data Availability

The data used to support the findings of this study are available from the corresponding author upon request.
